# In vitro molting of *Dirofilaria immitis* third-stage larvae derived from microfilariae collected from doxycycline-treated dogs

**DOI:** 10.1007/s00436-025-08506-z

**Published:** 2025-06-03

**Authors:** Yi Chu, Elyssa Campbell, Michael Dzimianski, Christopher C. Evans, Cassan Pulaski, Kaori Sakamoto, Andrew R. Moorhead

**Affiliations:** 1 Department of Infectious Disease, College of Veterinary Medicine, University of Georgia, Athens, GA USA; 2 Department of Population Health and Pathobiology, College of Veterinary Medicine, North Carolina State University, Raleigh, NC USA; 3 Department of Clinical Sciences, College of Veterinary Medicine, North Carolina State University, Raleigh, NC USA

**Keywords:** *Dirofilaria immitis*, *Wolbachia*, Doxycycline, Molting, Heartworm

## Abstract

*Dirofilaria immitis*, also known as canine heartworm, contains an endosymbiont, *Wolbachia*, in all life stages. The antibiotic, doxycycline, has been incorporated into heartworm treatment protocols to eliminate *Wolbachia*. Previous studies indicate that subsequent infection cannot be established using viable third-stage larvae (L3) developed from doxycycline-treated microfilariae (mf). The stages in which the development of larvae is impacted by doxycycline remain unknown. We examined the impact of doxycycline on the third-stage to fourth-stage larval molt, as it is the first molt of *D. immitis* after it invades the vertebrate host. Microfilaremic blood was collected weekly from *D. immitis–*infected dogs with or without doxycycline treatment at 10 mg/kg as recommended by the American Heartworm Society. Blood was collected weekly until the end of doxycycline treatment. The blood was used for L3 production and mf isolation. *Wolbachia* levels in mf and L3 were measured using real-time quantitative PCR. L3 were cultured in vitro for 9 days to assess whether molting occurred. The Fisher’s exact test and Bonferroni correction were used for statistical analysis. The molting of L3 from the doxycycline-treated groups did not show a significant difference compared to the L3 from the control group at weeks 0, 1, 2, 3, and 4. The *Wolbachia* levels in mf and L3 decreased starting from 7 days post-treatment and remained less than five percent of controls throughout the treatment. Doxycycline treatment can eliminate *Wolbachia* in both mf and subsequently developed L3. The molts of the mf to L3 in the mosquito and the L3 to L4 molt in vitro do not appear to be impacted by the reduction or elimination of *Wolbachia*.

**Supplementary Information:**

The online version contains supplementary material available at 10.1007/s00436-025-08506-z.

## Background

Heartworm disease (HWD), caused by the parasitic nematode *Dirofilaria immitis*, affects multiple animal species worldwide. As of 2023, HWD has been diagnosed in all 50 states of the United States of America (USA) (American-Heartworm-Society 2023). Infected dogs harboring circulating microfilariae (mf) can be reservoirs for subsequent infection, and more than 30 species of animals, including humans, can be infected by this parasite (Nelson 2023). Incidence rates of HWD in the southeastern USA indicated an overall increase of 21.7% with minimal change in the proportion of dogs receiving heartworm prevention between the years 2013 and 2016 (Drake & Wiseman 2018). The increase in heartworm incidence rates demands additional efforts control and treat the disease.

*Dirofilaria immitis* is a filarial parasite related to the human pathogens *Onchocerca volvulus*, *Brugia malayi*, and *Wuchereria bancrofti*. *Dirofilaria immitis* requires a vertebrate host and an invertebrate host to complete its life cycle. A mosquito takes a blood meal from an infected host containing circulating mf; the ingested mf then develop into third-stage infective larvae (L3) in the mosquito. When the mosquito takes another blood meal from a susceptible host, the L3 migrate to the mouth parts of the mosquito and enter the host through the bite wound created by the feeding activity (Abraham 1988; McCall et al. 2008). In the subsequent host, the L3 develop and molt into fourth-stage larvae (L4) three to 12 days after infection. The L4 eventually molt into immature adults and are present in the pulmonary artery by 120 days post-infection, where the adult male and female worms mate and produce mf (Abraham 1988; Kotani & Powers 1982; McCall et al. 2008). Transmission and the life cycle continue when another mosquito takes a blood meal from the infected host.

The American Heartworm Society (AHS) recommends using three doses of melarsomine dihydrochloride via intramuscular injection as the adulticidal treatment of HWD. Prior to the first dose of melarsomine, AHS recommends a 28-day course of doxycycline, a bacteriostatic agent belonging to the tetracycline family, at 10 mg/kg BID. Doxycycline can effectively reduce the bacterial endosymbiont *Wolbachia* in the parasite and subsequently reduce pathology and complications that may occur in the host following adulticidal treatment (Foster et al. 2005; Kramer et al. 2008; Nelson et al. 2017). Doxycycline has been shown to be effective against early infection of *D. immitis* in dogs by McCall et al. (2011) in a *Brugia pahangi* and *D. immitis* co-infection model. Experimental dogs were inoculated with 200 *B. pahangi* L3 and 50 *D. immitis* L3 via subcutaneous injection. There were no *D. immitis* and *B. pahangi* recovered at the end of the study (218–222 days post-infection) in the group that received doxycycline 0–29 days post-infection (dpi), which spans over the L3 and L4 molts of the parasites (McCall et al. 2011).

*Wolbachia* is a *Rickettsia*-like, intracellular, bacterial endosymbiont that is found in a variety of filarial nematodes that cause filariasis and dirofilariasis (Taylor et al. 2013). Though the roles of this endosymbiont require further investigation, some studies have provided insights into the functions of this bacterium, indicating that five metabolic biosynthetic pathways are only found in *Wolbachia* and not in its nematode hosts: heme, riboflavin, flavin adenine dinucleotide (FAD), glutathione, and nucleotide synthesis pathways (Darby et al. 2012; Foster et al. 2005). *Brugia malayi* does not harbor six out of seven genes required for heme de novo synthesis and lacks all five genes required for riboflavin biosynthesis. However, the de novo biosynthesis pathways of purines and pyrimidines are found in *Wolbachia* from *B. malayi*, which suggests that *Wolbachia* may support nucleotide synthesis for the host, especially in oogenesis and embryogenesis during which DNA synthesis is in high demand (Foster et al. 2005). The depletion of *Wolbachia* sterilizes several filarial nematode species, including *B. pahangi*, *B. malayi*, and *D. immitis*, which adversely impacts the production of mf (Bandi et al. 1999; Rao & Weil 2002).

*Wolbachia* has been suggested to be required for the molting activities in *B. malayi* and *B. pahangi* (Casiraghi et al. 2002; Quek et al. 2022). Casiraghi et al. (2002) assessed the impact of different administration times of tetracycline treatment on *B. pahangi* using the gerbil (*Meriones unguiculatus*) model focused on the fourth-stage to immature adult molt. The gerbils were infected with 100 *B. pahangi* L3 and were euthanized at 54 dpi. The outcome was evaluated based on the number of worms recovered and the levels of *Wolbachia* persisted. Their results indicate that tetracycline treatment impacted the *B. pahangi* L4 to immature adult molt in vivo. All groups of worm recovered showed a significant decrease in *Wolbachia* levels. The gerbils that received tetracycline before the molt of both sexes showed a reduced worm recovery. The gerbils received treatment after the male molted but before the molt of females had sex-ratio distorted worm recovery (Casiraghi et al. 2002).

McCall et al. (2014, 2023) investigated the ability of *D. immitis* L3 to complete normal development in naïve dogs when the inoculated L3 were produced from the blood of doxycycline-treated microfilaremic dogs (McCall et al. 2023, 2014). In the study published in 2014, the microfilaremic dogs received doxycycline at 10 mg/kg, BID, for 30 days. The blood was collected on days 73 to 77 post-doxycycline treatment and fed to mosquitoes. The authors then harvested L3 from the mosquitoes fed from the blood of the doxycycline-treated dogs, which were used to infect naïve dogs. Dogs infected with the L3 received blood examinations for mf and antigen beginning 5 months after inoculation until 302 dpi, when they were necropsied and examined for adult worms. No mf or detectable antigens were observed in the blood, and no live or dead adult *D. immitis* were recovered. Recovery of worms from dogs infected with L3 developed from the blood of untreated microfilaremic dogs averaged ten live male and 17 live female heartworms (McCall et al. 2014).

In the study published in 2023 (McCall et al. 2023), the authors investigated the ability of *D. immitis* L3 to develop from mf in the blood of dogs treated with doxycycline (10 mg/kg, once daily for 30 days) and ivermectin (6 µg/kg, given on days 0 and 30) at different time points throughout the treatment regimen. Blood was collected on days 22, 29, and 42 after the beginning of the treatment, and L3 produced by mosquitoes fed this blood were inoculated into naïve dogs (30–50 L3 per dog). The subsequently infected dogs were necropsied 163–183 dpi, and no adult worms were recovered from dogs that had received L3 developed from doxycycline/ivermectin-treated mf. However, dogs that received L3 developed from untreated mf had a recovery of 26–43 adult worms from each dog (McCall et al. 2023). However, no measurement of *Wolbachia* DNA in either mf or L3 was performed in the above two studies, leaving a gap in knowledge regarding the correlation between *Wolbachia* and the ability of L3 to develop into adult worms in subsequent hosts. We also do not know at which point the worms stopped developing in the host (L3 to L4 molt, L4 to immature adult molt), or whether the depletion of *Wolbachia* affected the immunomodulatory effect that the worms have against the host.

Our study explores the impact of doxycycline on mf and subsequently produced L3 through the measurement of *Wolbachia* DNA in mf and L3 at different times throughout the AHS-recommended doxycycline treatment duration of 28 days. Previous studies have demonstrated an inhibition of molting in *Brugia* spp. with tetracycline treatment (Casiraghi et al. 2002; Quek et al. 2022; Rao & Weil 2002; Smith & Rajan 2000), so we wanted our study to focus on the first molt after the *D. immitis* L3 enter the host. Due to limitations in the ability to track *D. immitis* L3 to L4 molting in vivo, we established a *D. immitis* in vitro culture/molting system to observe the impact of *Wolbachia* on molting.

## Method

**Fig. 1:**
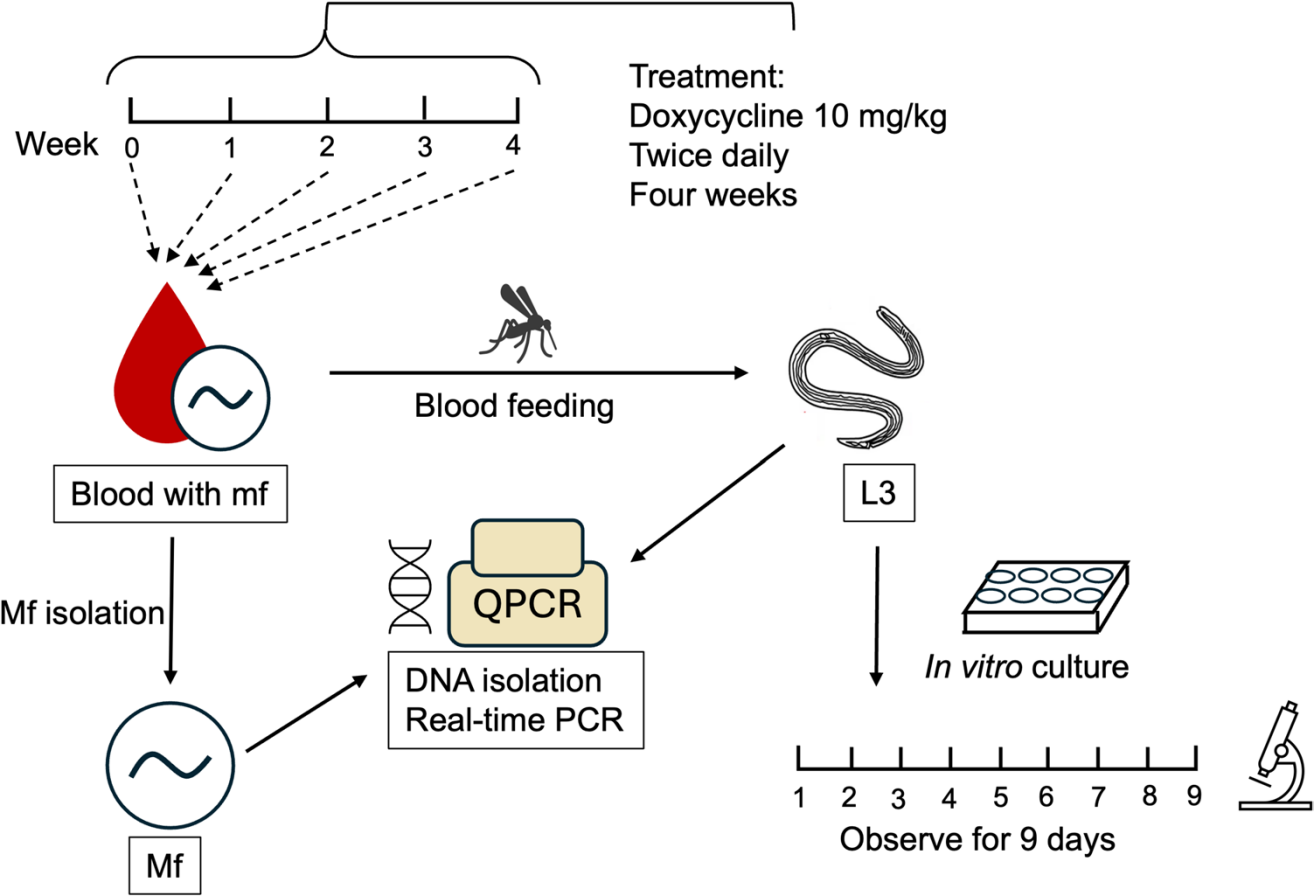
Experimental design flow chart. The animals received sham treatment or doxycycline treatment at 10 mg/kg for 28 consecutive days (4 weeks). The blood was collected weekly for L3 production and mf isolation. L3 were set up for in vitro culture and observed for 9 days. The *Wolbachia* levels were measured in mf and L3

A summary of the experimental design is shown in Fig. 1.

### Study animals

All experiments with purpose-bred dogs were performed according to the University of Georgia Institutional Animal Care and Use Committee guidelines using approved Animal Use Protocol A2022 04–006.

Three dogs were experimentally infected with 50 *D. immitis* Missouri (MO) isolate and housed in a specific pathogen-free environment. After the dogs became microfilaremic (9 months post-infection), they were randomly sorted into two groups: one dog served as the negative control, and two dogs received doxycycline hyclate (Epic Pharma, LLC, Laurelton, NY) orally at 10 mg/kg twice daily for 28 consecutive days. Blood was collected from the jugular vein before the start of doxycycline treatment and weekly after the treatment began.

### Microfilaria isolation from whole blood

The mf in blood were collected for DNA isolation and subsequent *Wolbachia* quantification. Whole blood was diluted with saponin solution, consisting of 0.2% saponin (Tokyo Chemical industry, Tokyo, JPN) and 0.85% sodium chloride (Sigma-Aldrich, St. Louis, MO) in distilled water at a 1:11 ratio (i.e., 3 mL blood with 33 mL solution). The mixture was incubated for 15 min at 37 °C for hemolysis. The hemolyzed sample was centrifuged at 850 × *g* for 10 min at room temperature, and the supernatant was discarded. The mf were washed twice by filling the tubes with phosphate-buffered saline (PBS) and repeating the centrifugation step. After the second wash, mf were resuspended with 10 mL PBS and quantified by averaging the count from two 10-µL aliquots and observing at 10X magnification via light microscopy. Each sample was diluted to approximately 1000–3000 mf per mL. Microfilariae were stored at − 80 °C until DNA isolation was performed.

### Mosquito feeding and L3 collection

Blood samples from three dogs were used for the production of L3 and *Wolbachia* DNA analysis in this study. Laboratory-raised *Aedes aegypti*, black-eyed, Liverpool strain were cultured and fed with blood collected from control and treatment groups (the blood from each animal was fed separately at each time point). Mosquitoes were harvested, and L3 were collected 15 days post-feeding per standard FR3 procedures (SOP numbers 8.2–8.4) (FR3, accessed 2024) with minor alterations. Briefly, mosquitoes were stunned at low temperatures, gently crushed in a mortar with a pestle, transferred to a 32-µm mesh sieve, quickly washed, and then soaked with 2% ciprofloxacin (Sigma-Aldrich, St. Louis, MO)-supplemented Hanks’ balanced salt solution (instead of Hanks’ balanced salt solution supplemented with penicillin–streptomycin as described in SOP 8.4). The L3 were manually quantified under light microscopy.

### DNA isolation and quantitative real-time PCR

DNA samples were isolated from mf (see additional file Table S2 for details) and L3 of the control and treatment groups. DNeasy Blood & Tissue kit (QIAGEN, Valencia, CA) was used for DNA isolation. All samples were processed according to the manufacturer’s instructions. Isolated DNA was stored at − 20 °C for qPCR analysis.

Quantitative PCR was performed to determine the ratio of *Wolbachia ftsZ* DNA to *D. immitis* 18S ribosome DNA using the primer set listed (Table 1). SsoAdvanced™ Universal SYBR® Green Supermix (Bio-Rad, Hercules, CA) with Bio-Rad CXF96 Touch Real-Time PCR Detection System was used. The reactions were designed based on the protocol provided with the SYBR Green Supermix mentioned above. Briefly, the reaction was started at 98 °C for 2 min, followed by 40 cycles of 95 °C for 15 s, 54 °C for 30 s, 60 °C for 30 s, signal detection, and melting curve generation (from 60 to 90 °C, at 0.5 °C interval). Primers were diluted to 500 nM for all reactions in each well, and the loading amount of DNA template ranged from 1 to 10 ng. Raw data were collected with CFX Maestro Software version 3.1.1517.0823 (Bio-Rad).

**Table 1:** Primers used for *Wolbachia* DNA quantification

Gene name		Primer sequence (5’–3’)	Reference
*D. immitis* 18S rRNA	Forward	TGAGAAACGGCTACCACATC	GenBank: AF036638
	Reverse	GATAACCGGCCTCATAGAGAAC	
*D. immitis Wolbachia FtsZ*	Forward	GCTGGTGCCTTACCTGATATT	GenBank: AJ495000 (Savadelis et al. 2018)
	Reverse	CCACCCATTCCTGCTGTTAT	

### In vitro molting assessment

Third-stage larvae were washed according to a protocol from the Zamanian lab at the University of Wisconsin—Madison (Mostafa 2024) (Protocols – parasitic nematodes – filarial nematodes – *Dirofilaria immitis* L3 to L4 molt) with a few changes. Briefly, we used a different media and antibiotic mix compared to the protocol mentioned above. L3 were transferred to a 1.5-mL microcentrifuge tube with 500 µL of wash media RPMI-1640 with L-glutamine (Lonza Bioscience, Walkersville, MD) supplemented with 100 U/mL penicillin and 100 µg/mL streptomycin (Penicillin–Streptomycin solution, Thermo Fisher Scientific, Waltham, MA), 10 µg/mL ciprofloxacin, and 100 µg/mL gentamycin (Sigma-Aldrich, St. Louis, MO) and centrifuged at 1000 × *g* for 10 min at room temperature. The concentrated L3 were transferred to new microcentrifuge tubes pre-filled with 500 µL washing media. The centrifugation and transfer steps were repeated twice. The L3 were transferred to a Petri dish containing culture media consisting of the washing medium supplemented with 10% heat-inactivated fetal bovine serum (FBS; Thermo Fisher Scientific, Waltham, MA). The L3 were examined under a dissecting microscope, and individual L3 were aliquoted to single wells of a 96-well plate. An additional 200 µL of culture medium was added to each well containing a single L3. The L3 were cultured in an incubator at 37 °C and 5% CO_2_. A pan of autoclaved water was placed in the incubator throughout the culture period.

An optical microscope was used to observe the L3 at 400X magnification for nine consecutive days. The presence of a fully detached cuticle was defined as a successful molt.

### Data analysis

The qPCR results were collected as *C*_t_ values. All *C*_t_ values were processed by CFX Manager software (Bio-Rad, Hercules, CA, USA, 3.1.1517.0823), and 2^−ΔΔCt^ (fold change) was calculated using the Livak method (Livak & Schmittgen 2001). The readouts for the *Wolbachia ftsZ* gene for each sample were normalized to the internal control (*D. immitis* 18S rRNA gene) and then to the control at each time point.

Fisher’s exact test was applied to determine the dependency of doxycycline treatment versus in vitro L3 to L4 molting rate. To calculate whether the molting event significantly differed between the control and treated groups, the Log-rank (Mantel-Cox) test was compared to the survival curves. Bonferroni correction was applied to both analyses.

## Results

### Quantitative real-time PCR

**Fig. 2:**
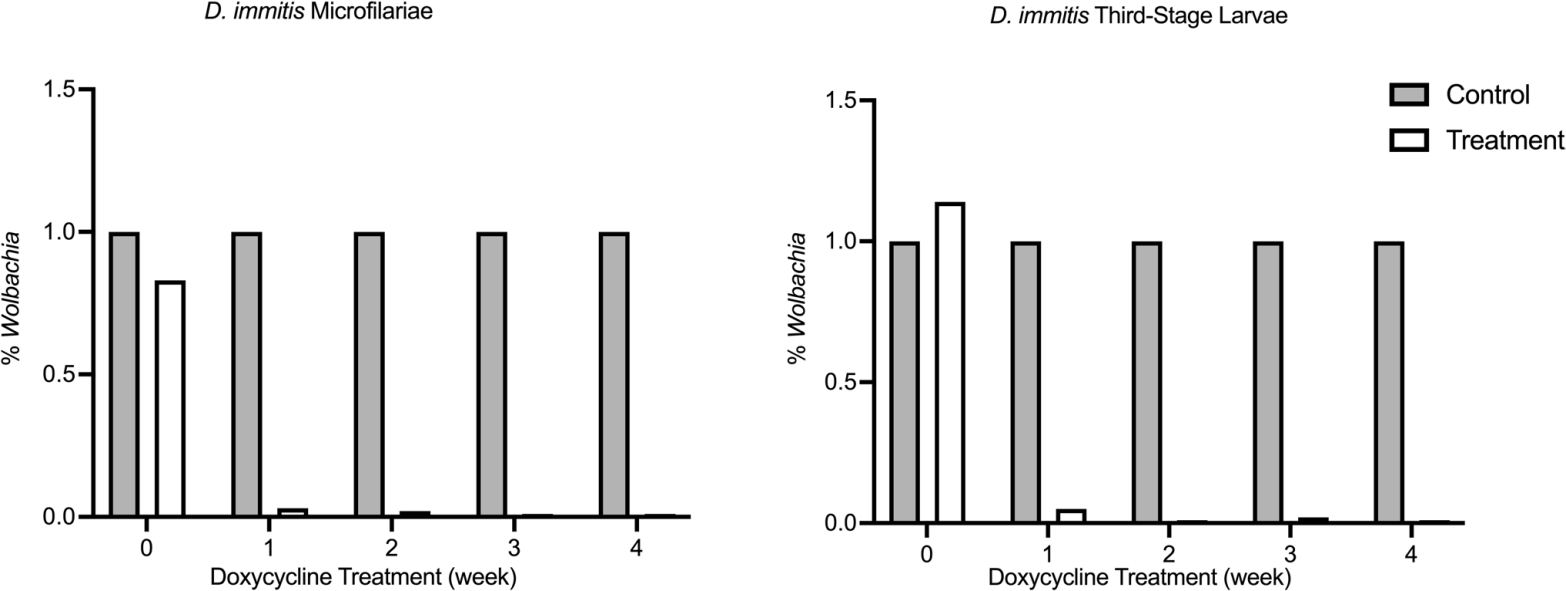
Fold-change *of Wolbachia* DNA in *D. immitis* mf and L3. *Wolbachia* DNA were measured using qPCR with ftsZ/Di 18 s rRNA DNA ratio in both mf (**A**) and L3 (**B**). The results indicate a substantial decrease after 7 days of doxycycline (< 5% of the control group) and remained at low levels throughout the treatment

The *Wolbachia* levels in mf and L3 demonstrated a similar trend (Fig. 2). Following 7 days of treatment, the *Wolbachia* levels in treatment groups decreased to < 5% of the control group in both mf and L3 and continued to decrease as the treatment progressed.

### In vitro L3 to L4 molting

We tested different culture conditions for in vitro molting with non-treated *D. immitis* L3. (additional file Text S1). Based on the observation (molting rate, death rate, and the time-to-molt event, data not shown), we decided that 200 µL RPMI-1640 media containing 10% FBS is sufficient to support the in vitro survival and molting of one *D. immitis* L3 for at least 9 days without media change, and the 9-day observation could include most of the molting events.

We defined a completed, successful molt by the identification of a fully detached cuticle in the culture plate. Using Fisher’s exact test, we analyzed whether the successful molting correlated with doxycycline treatment by examining the number of L3 that had molted by the end of the observation period. There was no significant difference in the number of L3 that molted between the control and the doxycycline-treated parasites, with the exception of week 2 (Fisher’s exact test, *p* = 0.008) (Table 2). The L3 that molted in week 2 from the control group reached a molting rate of 83.3%, while the molting rates of the other weeks were 31.3%, 72.9%, 51.2%, and 51% for weeks 0, 1, 3, and 4, respectively. The molting rate for the treatment group reached 60.8% on week 2, while the molting rates of the other weeks were 26.8%, 82.3%, 63%, and 64.6% for weeks 0, 1, 3, and 4, respectively.

**Table 2:** Number of molted control and doxycycline-treated L3

Doxycycline		Week 0		Week 1		Week 2		Week 3		Week 4	
		-	+	-	+	-	+	-	+	-	+
Molt	Yes	15	26	35	79	40	59	21	58	25	62
	No	33	71	13	17	8	38	20	34	24	34
*p*#		0.6954		0.1994		0.0076**		0.2517		0.1515	

#Fisher’s exact test

**Fig. 3:**
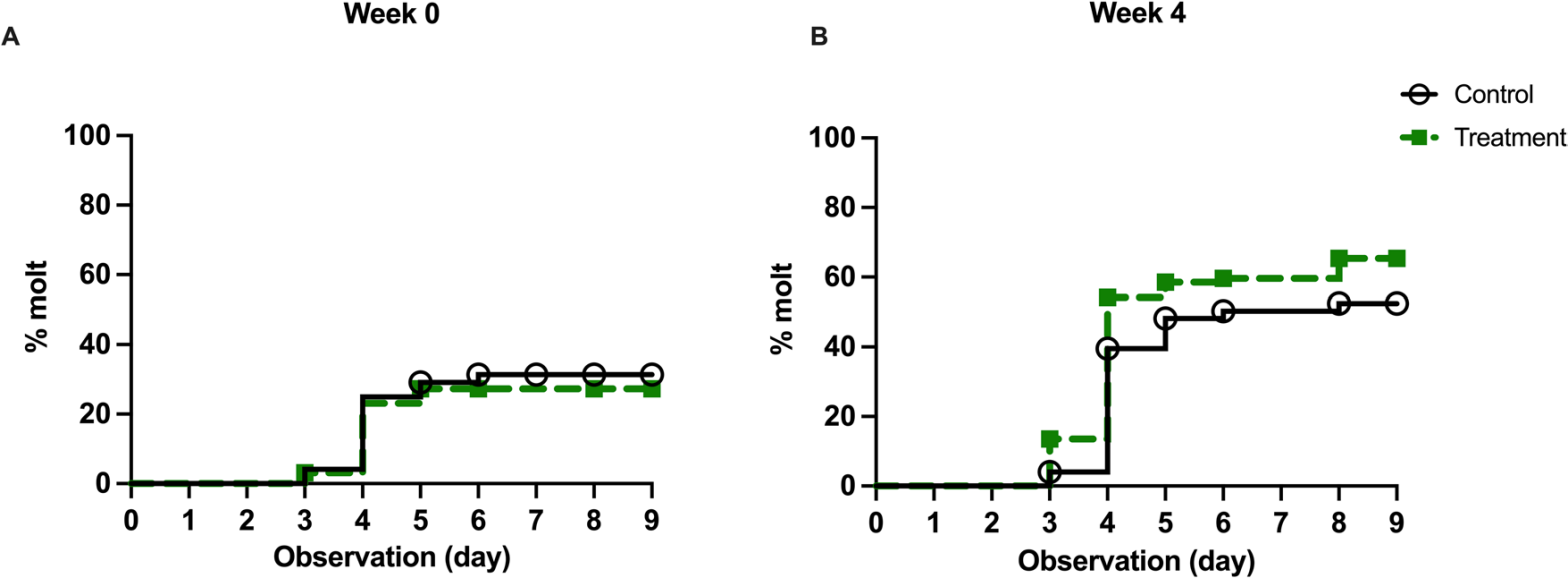
Time-to-molt events of *D. immitis* L3 through doxycycline treatment. The observation of a fully detached cuticle indicated the success of the molt event. Deaths of L3 that occurred before molt were excluded from the group. The percent molt represents the ratio of successfully molted L3 on a given day after the start of the culture to the total number of L3 included in the experiment. The doxycycline treatment in all weeks does not significantly impact the time-to-molt event

The time-to-molt event was analyzed by recording the day that the L3 molted and excluding any L3 that died before the observation endpoint. We did not observe any significant impact of doxycycline on the time-to-molt events. We used the log-rank (Mantel-Cox) test in Kaplan–Meier survival curves to compare molting activity based on doxycycline treatment. The molting pattern was not significantly affected by the doxycycline treatment (Mantel-Cox test; week 0, *df* = 1, *p* = 0.622; week 4, *df* = 1, *p* = 0.085) (Fig. 3).

## Discussion

Heartworm disease affects multiple hosts (e.g., felids, bears, ferrets, seals, sea lions, and humans) and is diagnosed in all continents except Antarctica (Nelson 2023). Doxycycline is an important component of heartworm treatment plan due to its effect on the bacterial endosymbiont *Wolbachia* (Bazzocchi et al. 2008; Taylor et al. 2013). Previous studies by McCall et al. (2014, 2023) (McCall et al. 2023, 2014) indicated that *D. immitis* L3 cultured from doxycycline-treated mf can develop into viable L3 in mosquitoes but cannot develop into adults in subsequent vertebrate hosts. Based on the results of these previous studies, we inferred that the removal of *Wolbachia* leads to the inability of *D. immitis* to complete its life cycle. However, the mechanism behind this remains to be determined. Hypotheses include that the inhibition of L3 to L4 molt, the inhibition of L4 to immature adult molt, and the depletion of *Wolbachia* lead to altered host immune reactions against the worms.

We focused on the L3 to L4 molt in this study, which occurs days 3–12 post-infection in the host (Kotani & Powers 1982). Due to the lack of an in vivo tracking method of L3, we evaluated and established an in vitro model and observed the L3 for nine consecutive days. We hypothesized that L3 developed from doxycycline-treated mf would be unable to molt to L4 in vitro. Our results failed to support our hypothesis. The results demonstrate that the amount of *Wolbachia* DNA in mf and L3 was reduced substantially 7 days (week 1) after the beginning of the AHS recommended 28-day (4 weeks) doxycycline treatment and remained < 5% of the control group throughout the treatment. However, the successful molting rate and time-to-molt event were not impacted (Table 2, Fig. 3) with the exception of the successful molting rate for week 2 (*p* = 0.0076) (Fig. S1). The significance of the molting rate at week 2 may be due to biological variation from different batches of L3. A previously published study (Reaves et al. 2018) on *Brugia malayi* mf also found significant biological variation between different batches of mf. In the study, researchers obtained *B. malayi* mf from the gerbils’ peritoneal cavity and co-cultured them with human neutrophils and monocytes to observe the killing effect. Despite no other treatment having been done, the results of the replicates of these experiments were significantly different. Our study focused on whether the absence of *Wolbachia* impacts the molting of *D. immitis* L3 to L4 in vitro, and the results that *Wolbachia*-depleted L3 could still finish the molt indicated a pronounced trend towards our conclusion—the molting was not impacted.

Our study investigated the impact of doxycycline without ivermectin at the preventive dose to reduce potential variations due to other factors besides *Wolbachia* elimination, which is microfilaricidal over time and may impact the general health of the mf (Bowman & Mannella 2011). Our data demonstrate that *Wolbachia* DNA is almost non-detectable in mf and L3 after doxycycline treatment. While *Wolbachia* DNA had been examined in mf in previous studies, it had not been examined in L3 derived from mosquitoes fed blood from doxycycline-treated dogs. These results suggest that the inability of mf from doxycycline-treated dogs to complete their life cycle in subsequent hosts is due to disruption in the life cycle at a developmental stage beyond the L3 molt.

Multiple studies have been performed examining the effect of tetracycline treatment on the molting of filarial nematodes, including *B. malayi*, *B. pahangi*,* O. volvulus*, and *D. immitis* (Albers et al. 2012; Quek et al. 2022; Rao et al. 2002; Smith & Rajan 2000)*.* Our data indicate that the presence of *Wolbachia* may not be required for *D. immitis* L3 to molt to L4 in vitro*.* Smith et al. (2000) examined the in vitro impact of tetracycline against the development of several filarial nematodes, and their results indicate inhibition of molting. They cultured *B. malayi* and *B. pahangi* with *Rhodotorula minuta* supplemented by arachidonic acid, while *D. immitis* was cultured without cellular coculture. Tetracycline (10 µg/ml) was added at the beginning of the culture (10 days for *Brugia* spp., 3 days for *D. immitis*). Tetracycline treatment inhibited *B. malayi* L3 to L4 molting by 93.9% of the controls on day 10, and the *D. immitis* L3 to L4 molt reached inhibition at 57.5% of the controls on day 3. They performed PCR to determine the amount of *Wolbachia* DNA in each sample, and samples collected from tetracycline-treated larvae still showed amplified bands on gel electrophoresis (Smith & Rajan 2000). There are several potential explanations for the discrepancy between their study and ours. The longer duration of treatment may explain the decrease of *Wolbachia* in our L3 samples. The timepoint we tested for *Wolbachia* DNA reduction was 7 days after the beginning of the treatment (week 1), while Smith et al. (2000) tested for *Wolbachia* 3 days post-treatment. The treatment conditions (in vitro vs. in vivo) could contribute to the difference in molting results. The actual medication used (tetracycline vs. doxycycline, with doxycycline being more lipophilic (Barza et al. 1975) and the endpoint of molting observation (3 days vs. 9 days) may also impact the outcomes.

There are some differences in L4 obtained from in vitro or in vivo molting. Marriott et al. (2023) developed an immunodeficient mouse model and obtained in vivo–developed L4 from inoculated L3 (Marriott et al. 2023). Alternatively, they cultured L3 in vitro with DMEM supplemented with only 10% FBS or with the addition of Madin-Darby canine kidney cells or rhesus monkey kidney epithelial cells in addition to the FBS. The authors compared the morphology of L4 as well as the amount of *Wolbachia* DNA in L4 obtained from mice 14 dpi and after 14 days of in vitro culture. The L4 cultured in vitro were significantly smaller compared to the L4 cultured in vivo. In vitro–derived L4 also presented with microscopic degenerative phenotypes, including malformed cuticle, hypodermis, buccal cavity, esophagus, and intestine (Marriott et al. 2023). We observed some deformed L3 during the in vitro culture process in both the control and treatment groups (data not shown). However, we could not rule out whether this was due to manipulation of the worms. The amount of *Wolbachia* DNA in the in vivo–derived L4 expanded 66-fold on average, while the in vitro–derived L4 did not display a notable change in *Wolbachia*. This could suggest that the host immune system, the nutrition that L3 could receive, as well as the host-parasite interaction, contribute to the fitness of the worms and their endosymbiont, *Wolbachia*, thus explaining the differences in the amount of *Wolbachia* DNA between in vivo and in vitro–derived larvae.

*Wolbachia* DNA in mf were measured weekly throughout the 28-day doxycycline treatment in a clinical study (Savadelis et al. 2018). Savadelis et al. (2018) studied eight dogs diagnosed with heartworm disease with doxycycline treatment at 10 mg/kg twice daily for 28 days and a monthly dosage of ivermectin/pyrantel (Heartgard® Plus, Boehringer Ingelheim, Duluth, GA). The dogs were brought to the clinic weekly for blood collection and qPCR was performed to detect the amount of *Wolbachia* DNA in mf at weeks 0, 1, 2, 3, and 4 post-treatment. This previous study showed that six out of eight dogs tested positive for *Wolbachia* DNA in mf at week 1. All dogs were cleared of *Wolbachia* DNA in the mf at the end of the study (Savadelis et al. 2018). This difference may be due to several variables. The authors defined *Wolbachia* DNA positivity as the qPCR result that could amplify *Wolbachia* DNA within 38 cycles of the reaction. In contrast, our qPCR looked at the relative changes in *Wolbachia* DNA compared to *D. immitis* DNA, which focused on the difference in *C*_t_ values between the *Wolbachia* gene and the *D. immitis* gene. Also, clinical studies have limitations in terms of animal conditions, the original burden of infections, and the compliance of pet owners. Those aspects may impact the efficacy of doxycycline treatment, thus leading to a delay in *Wolbachia* DNA reduction.

Answering the questions of whether the *D. immitis* L3 can also molt in vivo and exactly how doxycycline treatment inhibits *D. immitis* development in vivo requires further investigation. Tracking the L3 once they enter the host is time-consuming and cost-intensive (Kotani & Powers 1982). For our study, we optimized an in vitro *D. immitis* L3 to L4 molting system with nutrients supplied by only RPMI-1640 and heat-inactivated FBS, sufficient to support the overall survival for at least 9 days. Though widely used in cell culture, the components of FBS are complicated and may differ between manufacturers and batches. The genetic analysis revealed the potential metabolic role of *Wolbachia* in filarial nematodes, including the synthesis of heme and glutathione (Darby et al. 2012; Foster et al. 2005). Studies focused on FBS implied a likelihood of the existence of heme (Wagner et al. 2007), and glutamic acid and glutamine were found (Lee et al. 2023). We believe that FBS should not act as a replacement for *Wolbachia* and therefore interfere with the experimental outcome. The previous finding demonstrated that in vivo molting of tetracycline-treated *B. malayi* and *B. pahangi* was impaired (Casiraghi et al. 2002), indicating that *Wolbachia* contributes to molting in an environment that contains serum.

This system allows us to determine whether each cultured L3 can molt with fewer variables (i.e., cell viability if cell culture is involved). We demonstrated that doxycycline-treated *D. immitis* L3 can molt in vitro into L4. These results suggest that the inhibition of worm development in the host may occur after the L3 to L4 molt.

## Conclusion

Our research demonstrated that doxycycline reduces *Wolbachia* DNA levels in mf and subsequently derived L3 1 week after the start of the AHS-recommended 28-day treatment. Our research also suggests that under in vitro conditions, the depletion of *Wolbachia* via in vivo doxycycline treatment does not impact the successful molting rate or the time-to-molt event of *D. immitis* L3 to L4.

## Supplementary Information

Below is the link to the electronic supplementary material.

ESM 1 Supplementary file1 Time-to-molt events of D. immitis L3 through doxycycline treatment of weeks 1, 2, and 3. The observation of a fully detached cuticle indicated the success of the molt event. Deaths of L3 that occurred before molt were excluded from the group. The percent molt represents the ratio of successfully molted L3 on a given day after the start of the culture to the total number of L3 included in the experiment (PNG 227 KB)

High Resolution Image (TIF 636 KB)

Supplementary file2 Information of animals used in experiment (DOCX 17 KB)

Supplementary file3 Microfilariae isolation and DNA isolation (DOCX 19 KB)

Supplementary file4 In vitro culture condition testing (DOCX 104 KB)

## Data Availability

The data that support the findings of this study are available from the corresponding author upon reasonable request.
