# Supplementary figures and images for: In vitro molting of Dirofilaria immitis third-stage larvae derived from microfilariae collected from doxycycline-treated dogs
Source: Parasitol Res. 2025 Jun 3;124(6):59. doi: 10.1007/s00436-025-08506-z (PMC12133980; doi:10.1007/s00436-025-08506-z)

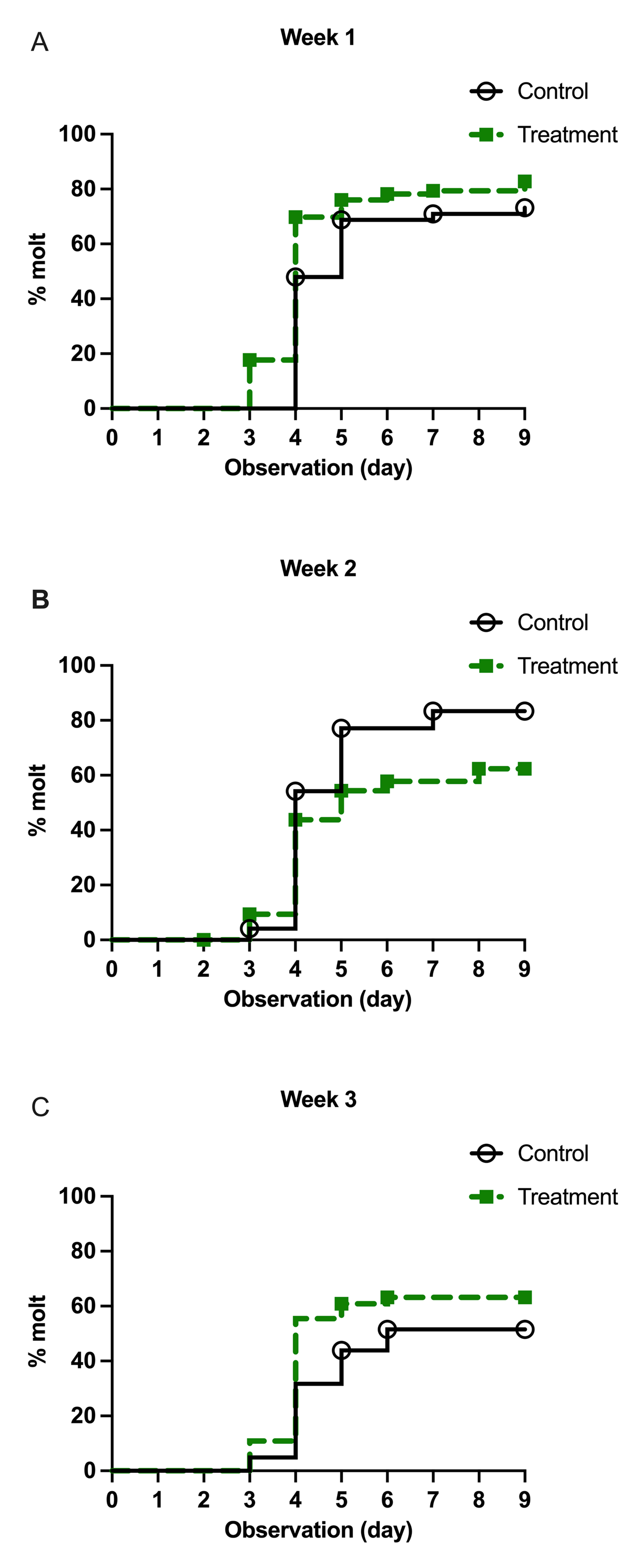

Supplement: Supplementary file 1 — Supplementary file1 Time-to-molt events of D. immitis L3 through doxycycline treatment of weeks 1, 2, and 3. The observation of a fully detached cuticle indicated the success of the molt event. Deaths of L3 that occurred before molt were excluded from the group. The percent molt represents the ratio of successfully molted L3 on a given day after the start of the culture to the total number of L3 included in the experiment (PNG 227 KB) [file 436_2025_8506_Fig4_ESM.png]

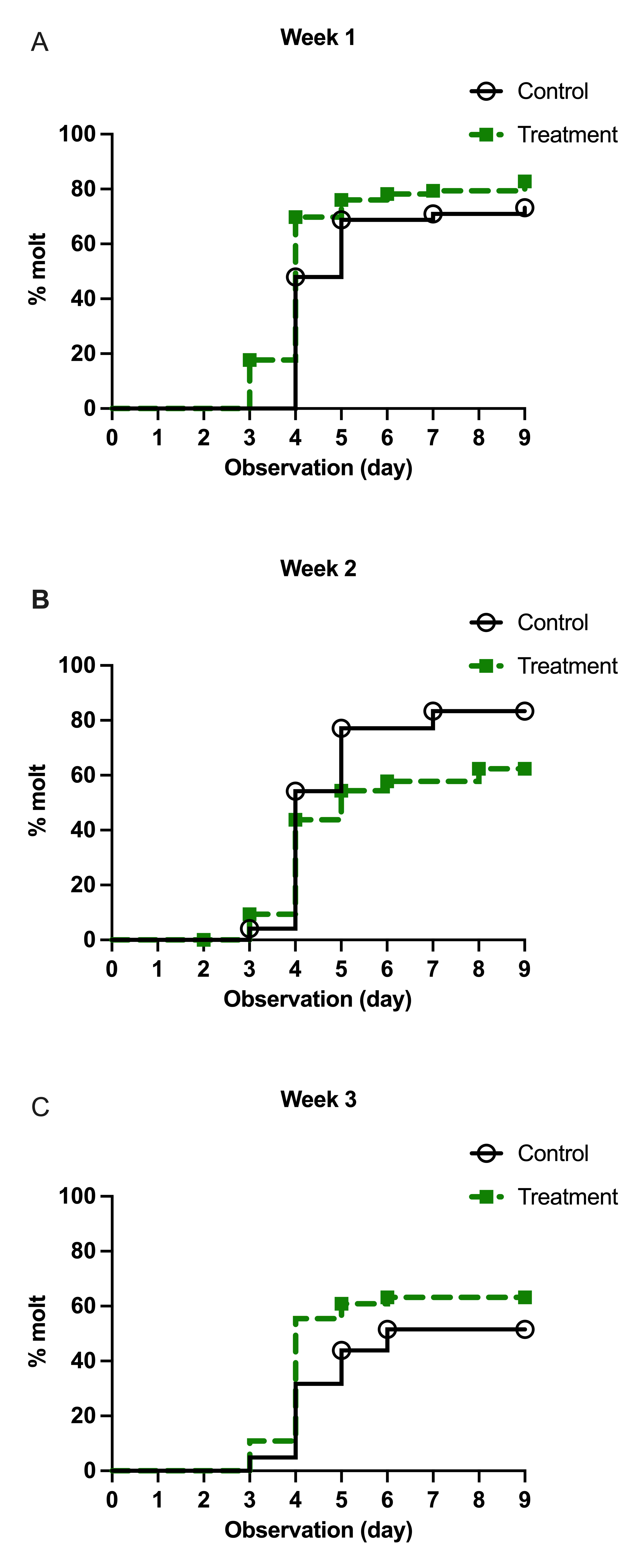

Supplement: Supplementary file 2 — High Resolution Image (TIF 636 KB) [file 436_2025_8506_MOESM1_ESM.tiff]
