# Supplementary material for: In vitro molting of Dirofilaria immitis third-stage larvae derived from microfilariae collected from doxycycline-treated dogs
Source: Parasitol Res. 2025 Jun 3;124(6):59. doi: 10.1007/s00436-025-08506-z (PMC12133980; doi:10.1007/s00436-025-08506-z)
Supplement: Supplementary file 3 — Supplementary file2 Information of animals used in experiment (DOCX 17 KB) [file 436_2025_8506_MOESM2_ESM.docx]

| Animal No. | Sex | Age (at inoculation) | Microfilaremia | Doxycycline |
| --- | --- | --- | --- | --- |
| 1 | Male | 1 year 4 months | 9 months PI | + |
| 2 | Male | 1 year 4 months | 9 months PI | + |
| 3 | Male | 1 year 4 months | 9 months PI | - |
