# Supplementary material for: In vitro molting of Dirofilaria immitis third-stage larvae derived from microfilariae collected from doxycycline-treated dogs
Source: Parasitol Res. 2025 Jun 3;124(6):59. doi: 10.1007/s00436-025-08506-z (PMC12133980; doi:10.1007/s00436-025-08506-z)
Supplement: Supplementary file 4 — Supplementary file3 Microfilariae isolation and DNA isolation (DOCX 19 KB) [file 436_2025_8506_MOESM3_ESM.docx]

| Collection time | Animal Id | mf amount (total) | mf for DNA isolation | DNA concentration ng/uL |
| --- | --- | --- | --- | --- |
| Week 0 | 1 | 20000 | 2,000 | 4 |
|  | 2 | 25000 | 2,000 | 6.6 |
|  | 3 | 5000 | 1,000 | 19.6 |
| Week 1 | 1 | 13000 | 770 | 63.6 |
|  | 2 | 6000 | 840 | 21.3 |
|  | 3 | 30250 | 770 | 16.9 |
| Week 2 | 1 | 28400 | 4000 | 11.1 |
|  | 2 | 24400 | 3600 | 10.2 |
|  | 3 | 36100 | 2600 | 10.6 |
| Week 3 | 1 | 12000 | 2400 | 14.3 |
|  | 2 | 8500 | 1600 | 9.6 |
|  | 3 | 8250 | 1700 | 16.7 |
| Week 4 | 1 | 2500 | 500 | 1.2 |
|  | 2 | 8400 | 2000 | 2.8 |
|  | 3 | 3500 | 300 | 1 |
