# Supplementary material for: In vitro molting of Dirofilaria immitis third-stage larvae derived from microfilariae collected from doxycycline-treated dogs
Source: Parasitol Res. 2025 Jun 3;124(6):59. doi: 10.1007/s00436-025-08506-z (PMC12133980; doi:10.1007/s00436-025-08506-z)

***In vitro* culture condition testing**

1. Objective: Determine the optimal FBS concentration and observation length.

FBS concentration tested:10%, 20%.

Result:

| Observation (day) | 1 | 2 | 3 | 4 | 5 | 6 | 7 | 8 | 9 | 10 | 11 | 12 | 13 | 14 | 15 | 16 |
| --- | --- | --- | --- | --- | --- | --- | --- | --- | --- | --- | --- | --- | --- | --- | --- | --- |
| 10% FBS molting rate | 0.00 | 0.00 | 0.00 | 22.73 | 59.09 | 59.09 | 68.18 | 72.73 | 77.27 | 77.27 | 77.27 | 77.27 | 77.27 | 77.27 | 81.82 | 81.82 |
| 20% FBS molting rate | 0.00 | 0.00 | 0.00 | 27.27 | 63.64 | 68.18 | 68.18 | 68.18 | 72.73 | 72.73 | 72.73 | 72.73 | 72.73 | 72.73 | 72.73 | 72.73 |
| 10% FBS death rate | 0.00 | 0.00 | 0.00 | 0.00 | 0.00 | 4.55 | 4.55 | 4.55 | 4.55 | 4.55 | 4.55 | 13.64 | 40.91 | 59.09 | 81.82 | 81.82 |
| 20% FBS death rate | 0.00 | 0.00 | 0.00 | 4.55 | 4.55 | 9.09 | 9.09 | 13.64 | 13.64 | 13.64 | 18.18 | 31.82 | 31.82 | 40.91 | 59.09 | 72.73 |


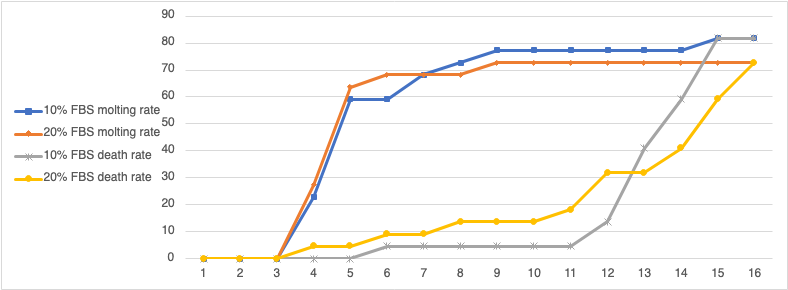


1. Objective: Determine if multiple L3 cultured in one well impact molting.

Condition tested: One L3 per well vs. 2 – 7 L3 per well of the 96-well plate. All cultures were cultured with 10% FBS and observed for nine days.

Result:

| Observation (day) | 1 | 2 | 3 | 4 | 5 | 6 | 7 | 8 | 9 |
| --- | --- | --- | --- | --- | --- | --- | --- | --- | --- |
| Single L3 | 0.00 | 0.00 | 0.00 | 26.47 | 61.76 | 70.59 | 70.59 | 70.59 | 79.41 |
| Multiple L3 | 0.00 | 0.00 | 0.00 | 25.00 | 56.82 | 63.64 | 64.77 | 65.91 | 69.32 |


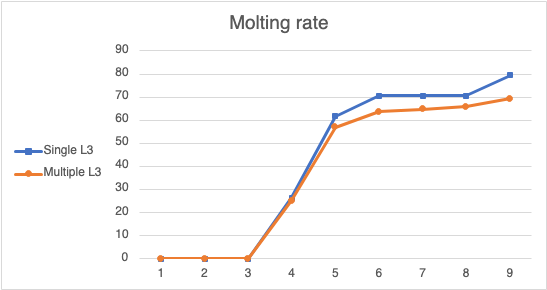


1. Objective: Determine if Matrigel facilitates molting.

Condition tested: 15 µL or 30 µL Matrigel (Corning, catalog #354234) in one well of the 96-well plate. All cultures were cultured at 10% FBS, one L3 per well, and observed for nine days.

Result:

| Observation (day) | 1 | 2 | 3 | 4 | 5 | 6 | 7 | 8 | 9 |
| --- | --- | --- | --- | --- | --- | --- | --- | --- | --- |
| Control | 0.00 | 0.00 | 0.00 | 26.47 | 61.76 | 70.59 | 70.59 | 70.59 | 79.41 |
| 15 µL Matrigel | 0.00 | 0.00 | 0.00 | 36.67 | 50.00 | 50.00 | 53.33 | 63.33 | 63.33 |
| 30 µL Matrigel | 0.00 | 0.00 | 0.00 | 46.67 | 60.00 | 66.67 | 66.67 | 66.67 | 66.67 |


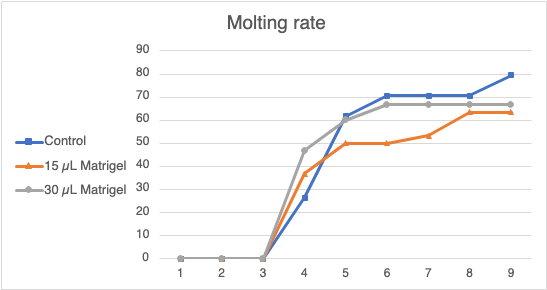

Supplement: Supplementary file 5 — Supplementary file4 In vitro culture condition testing (DOCX 104 KB) [file 436_2025_8506_MOESM4_ESM.docx]
